# Pharmaceutical Process

**Published:** 1805-08-01

**Authors:** 


### Pharmaceutical Process.

### *Goetling's improved Preparation of Kermes Mineral.*

REDUCE separately to powder, and afterwards mix sixteen parts of crude antimony, twenty-four parts of purified potash, and three parts of flowers of sulphur; introduce the mixture into a crucible, and let it enter into complete fusion. After it has cooled, pulverize the mass, and boil it for half an hour in one hundred and twenty-eight parts of water; filter it while boiling through a thick cloth, letting it run into an earthen pan containing one hundred and fifty-six parts of water, and leave it exposed to the air, in a shallow vessel, for two or three days, or until particles of a bright orange colour appear on its surface. Afterwards decant the liquid, wash the deposit in a large quantity of water; then remove it on a filter, and complete the edulcoration; when this is done, dry it by a gentle heat.

This process yields twelve or fourteen parts of Kermes mineral of a fine reddish brown colour; the whole quantity of antimony, except a trifling residuum of extraneous matter, is dissolved and converted into Kermes, and only a very small quantity remains in the decanted liquor, in the form of golden sulphur.

